# Mechanical Properties of a Supramolecular Nanocomposite Hydrogel Containing Hydroxyl Groups Enriched Hyper-Branched Polymers

**DOI:** 10.3390/polym13050805

**Published:** 2021-03-06

**Authors:** Wenjin Xing, Amin Jamshidi Ghahfarokhi, Chaoming Xie, Sanaz Naghibi, Jonathan A. Campbell, Youhong Tang

**Affiliations:** 1 College of Science and Engineering, Flinders University, Clovelly Park, Adelaide, SA 5042, Australia; wenjin.xing@flinders.edu.au (W.X.); amin.jamshidighahfarokhi@flinders.edu.au (A.J.G.); sanaz.naghibi@flinders.edu.au (S.N.); 2 Institute for NanoScale Science and Technology, Flinders University, Bedford Park, Adelaide, SA 5042, Australia; jonathan.campbell@flinders.edu.au; 3 Key Lab of Advanced Technologies of Materials, Ministry of Education, School of Materials Science and Engineering, Southwest Jiaotong University, Chengdu 610031, China; xie@swjtu.edu.cn

**Keywords:** toughening, fracture, nanocomposite hydrogel, supramolecular interactions, fractocohesive length

## Abstract

Owing to highly tunable topology and functional groups, hyper-branched polymers are a potential candidate for toughening agents, for achieving supramolecular interactions with hydrogel networks. However, their toughening effects and mechanisms are not well understood. Here, by means of tensile and pure shear testings, we characterise the mechanics of a nanoparticle–hydrogel hybrid system that incorporates a hyper-branched polymer (HBP) with abundant hydroxyl end groups into the matrix of the polyacrylic acid (PAA) hydrogel. We found that the third and fourth generations of HBP are more effective than the second one in terms of strengthening and toughening effects. At a HBP content of 14 wt%, compared to that of the pure PAA hydrogel, strengths of the hybrid hydrogels with the third and fourth HBPs are 2.3 and 2.5 times; toughnesses are increased by 525% and 820%. However, for the second generation, strength is little improved, and toughness is increased by 225%. It was found that the stiffness of the hybrid hydrogel is almost unchanged relative to that of the PAA hydrogel, evidencing the weak characteristic of hydrogen bonds in this system. In addition, an outstanding self-healing feature was observed, confirming the fast reforming nature of broken hydrogen bonds. For the hybrid hydrogel, the critical size of failure zone around the crack tip, where serious viscous dissipation occurs, is related to a fractocohesive length, being about 0.62 mm, one order of magnitude less than that of other tough double-network hydrogels. This study can promote the application of hyper-branched polymers in the rapid evolving field of hydrogels for improved performance.

## 1. Introduction

Due to a large volume fraction of water in the polymer network, most conventional hydrogels exhibit poor mechanical performance, greatly confining their application scope. For example, these compliant, weak, low-toughness, and notch-sensitive hydrogels cannot be used as supporting base materials for flexible bioelectronics [1,2,3], functional materials for externally controlled soft actuators [4,5], repeatedly loaded artificial muscles [6,7], and heavy load-bearing tissue scaffolds and replacements in tissue engineering [8,9]. To boost their practical potential, many synthetic superior hydrogels have been designed based on various introduced energy dissipation mechanisms [10,11]. Most noticeable tough hydrogels are double-network (DN) hydrogels, comprised of a rigid and brittle first network, and a soft and ductile second network covalently cross-linked [12,13]. The first network, either covalently or noncovalently cross-linked, serves as a sacrificial network, whose breakage into fragments can consume elastic energy; however, the second network maintains the basic shape under large deformation, even in the presence of macroscopic cracks. In this way, the alginate/polyacrylamide (PAAm) DN hydrogels could achieve exceptional mechanical behaviours with tensile strength of 160 kPa and fracture toughness of 9000 J/m${}^{2}$ [14].

Another popular family of design schemes for strengthening and toughening hydrogels is to consider supramolecular chemistry. Such examples include hydrophobic associations [15,16], inonic interactions [17,18], electrostatic attractions [19], hydrogen bonding [20,21,22], and host–guest interactions [23]. The various noncovalent supramolecular interactions endow hydrogels of supramolecular polymer networks with outstanding stretchability, strength, and toughness. Furthermore, these dynamic reversible bonds enable fast self-recovery under mild conditions [24,25].

A supramolecular elastomer or hydrogel can be fabricated by incorporating hyper-branched polymers (HBPs) into the backbone matrix of another polymer [26,27]. HBPs are highly branched dendritic macromolecules with many end groups [28]. Owing to their unique chemical properties such as high solubility, low viscosity, and a large free volume [29], HBPs have been successfully applied in many specific areas such as coatings [30] and biomedical treatments [31]. Benefiting from easy fabrication, abundant repeating units, and adaptable structures, HBPs are also prospective reinforcement material candidates for achieving hydrogels of high strength and toughness through supramolecular chemistry [32,33]. Polymer chains of hydrogels are able to form supramolecular cross-links with the structure of HBPs at their repeating units [34].

HBPs are generally synthesised using a stepwise process and tend to linearly increase in diameter, adopting a more globular shape with increasing generation. The term of generation refers to the number of repeated branching cycles during synthesis of a HBP [35,36]. Moreover, the repeating units are arranged in layers around the core, each layer representing one generation of the HBP [37]. Increasing generations suggest more functional reactive sites/groups arranged in the layers. For example, in this study the adopted HBPs of generation 2, 3 and 4 have 16, 32 and 64 hydroxyl groups on their shell, respectively. The shell of a HBP as a modifier could be functionalised with distinct functional groups for specific purposes, e.g., optimising toughening effects in the brittle epoxy resin [38,39,40].

In toughened hydrogels, crack blunting is typically observed, which seems to be relevant to large deformation [41,42]. The blunted crack geometry somewhat mitigates the stress concentration at the crack tip, thus making the hydrogel tough. However, in the study by Luo et al. [43], the authors showed that the blunting was more closely related to the condition where the fracture stress is larger than the modulus, than to large deformation. When defects or notches are small enough, the tough hydrogel is still capable of withstanding large deformation without any crack propagation. To elucidate such an effect of notch insensitivity, Chen et al. [44] proposed the concept of fractocohesive length for stretchable materials, quantified as the ratio of fracture toughness (energy) to work of fracture. This critical length scale can be applied to hydrogels for the approximate prediction of the transition from flaw-insensitive to flaw-sensitive fracture, and also the evaluation of the size of fracture process zone [45]. Yang et al. [46] revealed that inherent network imperfection in the synthesised polyacrylamide hydrogel gives rise to notch insensitivity and enhanced fracture toughness by orders of magnitude, compared to that of an ideally perfect polymer network.

Understanding the mechanical and fracture properties of tough hydrogels, which are based on the concept of supramolecular chemistry, has huge scientific significance and practical implications. In this work, we comprehensively investigate mechanics of a hybrid hydrogel system, comprising a building block of polyacrylic acid (PAA), and a specific type of HBP with many hydroxyl end groups. The choice of this type of HBP is based on the existence of abundant hydroxyl groups on the HBP structure (shell) that can in theory develop hydrogen bonding with the carboxylic groups on the long PAA chains, thus strengthening and toughening the hydrogel [22]. Three generations with a similar topology but hydroxyl groups of different numbers on each molecule were compared. Instead of designing a prominent tough hydrogel, the main objective of this work is to explore how different HBP generations serve in improving mechanical properties and fracture behaviour. The nature of HBP-induced supramolecular interactions is determined in an inverse manner mainly by means of mechanical testing.

## 2. Experimental Section

### 2.1. Hydrogel Preparation

We prepared the hydrogels by in-situ, free-radical and one-step polymerisation. Acrylic acid monomer (AA), hyperbranched bis-MPA polyester-32(or 16,64)-hydroxyl (HB), ammonium persulfate (APS), and N,N${}^{\prime }$-methylenebis (acrylamide) (MBA) were purchased from Sigma-Aldrich (Sydney, Australia) and used without further purification. Sodium hydroxide (NaOH) was supplied by Chem-Supply (Adelaide, Australia). Milli-Q water at room temperature was used as solvent.

To prepare the PAA and hybrid hydrogels, we followed the method described by Yu et al. [32]. For PAA hydrogels, 3.7 g AA was mixed with 3.5 cm${}^{3}$ Milli-Q water at room temperature. A 0.45 g cm${}^{-3}$ NaOH solution was made by dissolving 1.63 g sodium hydroxide powder in 3.62 g Milli-Q water, cooled in an ice-water bath for 10 min. Then, NaOH solution was drop-wise added into the AA solution under magnetic stirring for 15 min to fully neutralise the AA monomer. Subsequently, we added APS of 2.2 wt% of AA as thermal initiator, and MBA of 0.67 wt% of AA as cross-linker, into the mixture, followed by vigorous shaking with an IKA Vortex 3 shaker (Sigma-Aldrich, Sydney, Australia) to ensure homogeneity. The final mixture was poured into a culture dish of 90 mm in diameter and then cured by heating in a laboratory oven under a constant temperature of 60${}^{\circ }$ for 1 h. After heating, the synthesised PAA samples were peeled from the culture dish and stored in sealed plastic bags in the fridge, waiting for testing after three days.

**Figure 1:**
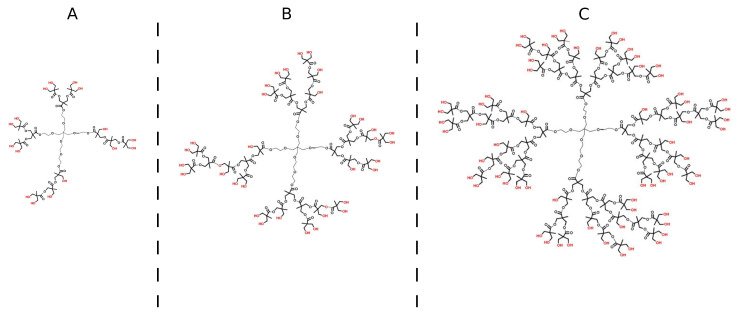
The molecular structures of hyper-branched bis-MPA polyester-16-hydroxyl polymer, generation 2 (**A**), hyper-branched bis-MPA polyester-32-hydroxyl polymer, generation 3 (**B**), and hyper-branched bis-MPA polyester-64-hydroxyl polymer, generation 4 (**C**).

**Figure 2:**
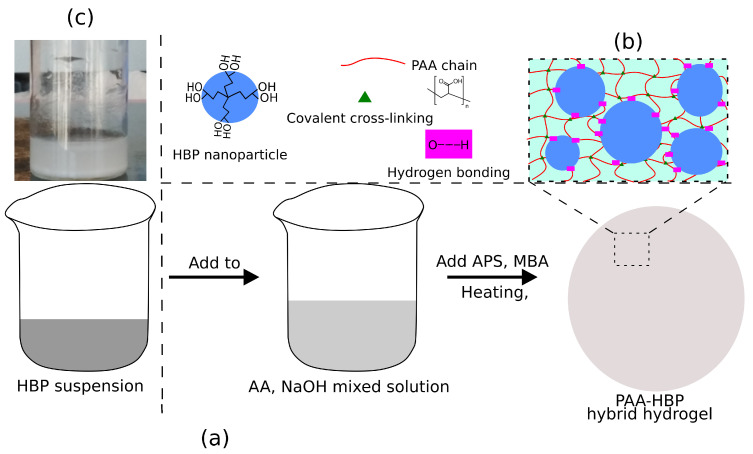
Schematic protocol for preparation of the hybrid hydrogel of PAA and HBP. (**a**) Facile preparation procedure. (**b**) Chemical and physical cross-linking inside the polymer network. (**c**) Photograph of HBP3 solution (0.05 g cm${}^{-3}$), forming a colloidal system.

Three generations of HBP with 16, 32, and 64 hydroxyl groups on each molecule were termed the second, third, and fourth generation, respectively. Their molecular structures are provided in Figure 1. For preparing hybrid hydrogels, a schematic protocol is shown in Figure 2a. For a weight content of 14% HBP in the total mass of AA and HBP, a suspension with 0.6 g HBP powder was first prepared, and then was added to the mixed solution of NaOH and 3.7 g AA, followed by vigorous shaking. The remaining operations were the same as those for PAA hydrogels. It should be noted that the polymerisation process of PAA hydrogels was not affected by the addition of HBPs, due to their low viscosity.

The as-prepared hybrid hydrogels were coded as PAH*m*–*n*, where *m* refers to the used generation of HBP, and *n* the weight percentage (×100) of HBP in the total mass of AA monomer and HBP powder. It is worth mentioning that the HBP substance forms a colloidal system in deionised water as shown in Figure 2c, whereas it can fully dissolve in the presence of PAA solution which is a polar solvent.

### 2.2. Scanning Electron Microscopic Study

For the morphological observation of hydrogels, scanning electron microscopy (SEM) (Inspect F50, FEI, Tokyo, Japan) was employed. The hydrogel samples were freeze-dried and then coated with platinum to provide a conductive environment. The spatial resolution was set as 500 μm.

### 2.3. Spectroscopic Analysis

To investigate possible interactions between the PAA polymer and HBP molecules, we examined the Fourier transform infrared (FTIR) absorption spectra of the HBP3 powder, freeze-dried PAA hydrogel, and freeze-dried hybrid hydrogel, using FTIR spectroscopy (PerkinElmer Spectrum 400 spectrometer, PerkinElmer, Waltham, MA, USA). Each sample was mounted onto the central crystal plate. FTIR spectra were collected by 64 scans from 500 to 4000 cm${}^{-1}$ with a resolution of 4 cm${}^{-1}$ in the absorbance mode.

### 2.4. Mechanical Testing

To quantify the basic mechanical properties for as-prepared hydrogels, uniaxial tensile testing was conducted, at room temperature, using universal testing equipment (Instron 5960, Norwood, MA, USA) with a load-cell of capacity 500 N. The cross-head speed was kept constant as 10 mm/min, if not otherwise mentioned. Displacement and load values were recorded from the testing system. Rectangular samples were tested with the effective length between rigid clamps specified as 10 mm. The sample width was fixed at 20 mm and thickness, about 1.2–1.6 mm, was measured with a digital caliper for each sample. The nominal stress σ was estimated from the tensile force divided by the cross-section area of the sample in the initial configuration. The stretch λ was evaluated from the current gap between clamps divided by the effective length. For each measurement, three test trials were performed to enhance the reliability of results. All the data represent the mean and standard deviation of three experimental results.

### 2.5. Fracture Toughness Measurement

In general, fracture toughness is defined as the energy required to extend the crack by a unit area. Classic pure shear tests were utilised to measure fracture toughness of the as-prepared hydrogels [14,47,48]. Two rectangular samples of the identical dimensions of 50 mm in width, 10 mm in height, and 1.2–1.6 mm in thickness, were prepared with one of them precut with a razor blade, to form a crack of 20 mm long, on the sample edge along the middle line between the clamps. A vertical red line of approximate thickness of 0.5 mm was marked through the crack tip on the surface of the precut sample, with a red ball-point pen to help to visualise the crack initiation. The sample containing a crack was pulled at a low loading speed and the critical stretch $\lambda_{c}$ was identified at the moment when the crack tip just crosses the thin red line. The fracture toughness, denoted by *G*, is expressed as

(1) $$G=W(\lambda_{c})\cdot H$$

where *H* is the sample height (i.e., 10 mm), and W(λ) represents the energy release rate, evaluated by the area below the stress–stretch curve of the intact sample up to the stretch of λ.

### 2.6. Hysteresis Measurement

To further demonstrate the capability of energy dissipation for PAH hybrid hydrogels, two consecutive loading-unloading cycles with different maximum stretches were applied under tension. For each loading–unloading cycle, a fresh hydrogel sample was used. The area enclosed by the loading and unloading curves measures the energy dissipated during a cycle of loading and unloading. A hysteresis coefficient was defined by the ratio $W_{D}/W$ of the energy dissipated $W_{D}$ to the work done *W*, both of which could be obtained from the stress–stretch curves [49].

### 2.7. Viscoelastic Behaviours

Viscoelastic responses of hydrogels were determined by dynamic mechanical analysis (DMA) using a frequency sweep of 0.1–50 Hz under compression at a strain of 0.5% and at a temperature of 20${}^{\circ }$. Storage modulus, loss modulus, and tan(δ) (loss modulus/storage modulus) were recorded by the analyser (TA Instruments DMA Q800, TA Instruments, New Castle, DE, USA).

### 2.8. Self-Healing Tests

To examine the self-healing capability of the hybrid hydrogel, two cycles of loading-unloading with different in-between holding intervals were performed under tension. The maximum stretch $\lambda_{max}$ was specified as 3.5. The hysteresis recovery was defined as the ratio between the first and second hysteresis coefficients, and the strength recovery as the ratio between the first and second strengths.

### 2.9. Finite Element Modelling

To gain deep insights into the in-plane deformation behaviour and blunting scenario for the PAA and hybrid hydrogels, numerical simulations were performed on the pure shear fracture test, with the finite element method (FEM) in the commercial software, ABAQUS. The Neo-Hookean and Mooney–Rivlin material models were utilised as hyper-elastic constitutive models for the PAA and hybrid hydrogels, respectively. Good matches between the tensile test data and fitted constitutive models were obtained, as shown in Figure S1 (ESI). In the FEM model, to avoid stress singularity, a blunt tip with a radius of 0.1 mm, was considered in the geometry. The sheet of hydrogel was discretised with plane stress elements (CPS4R), whose size was sufficiently small near the crack tip, in order to capture local strain gradients. Only one half of the sheet was modelled to reduce computational costs, due to the symmetric loading and geometry.

## 3. Results and Discussion

In this section, results are presented in sequence, regarding the characterisations with instruments (Section 3.1), the comparison of mechanical properties (Section 3.2) and fracture toughness (Section 3.3) among the hybrid hydrogels of three-generation HBPs, and the hysteresis and self-healing properties (Section 3.4). A discussion about the toughening mechanism of the hybrid hydrogel, crack-tip blunting scenario and fracture process is placed in Section 3.5.

### 3.1. Characterisations of Hybrid Hydrogels of PAA and HBP

By measurement, the as-prepared hydrogels contained a moderate water content of about 50% of the total weight. In the presence of water, the HBP molecules develop into nanoparticles of varying sizes as suggested by our previous transmission electron microscopy study [50]. These nanoparticles would generate supramolecular interactions with the backbone network of PAA cross-linked by covalent bonds.

**Figure 3:**
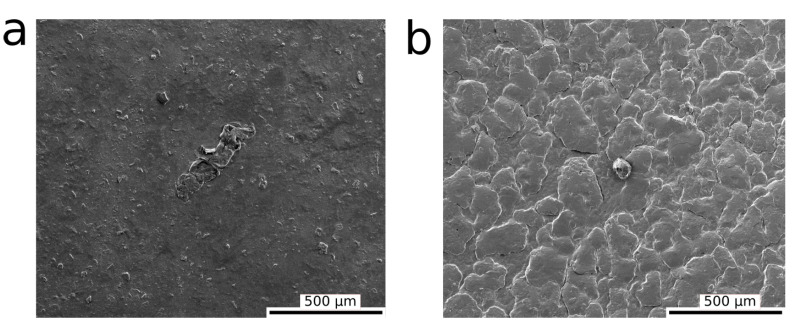
SEM images showing the surface morphology of hydrogels: (**a**) PAA, and (**b**) PAH3 (14 wt% HBP3), at a spatial resolution of 500 μm.

Figure 3 shows the morphological differences between the PAA hydrogels with and without the addition of HBP, at the microscale using SEM. A dense and smooth surface was observed for PAA hydrogel (Figure 3a), whereas the hybrid hydrogel of PAA and HBP displayed a relatively rough and wavy surface (Figure 3b), indicating potential physical interactions.

**Figure 4:**
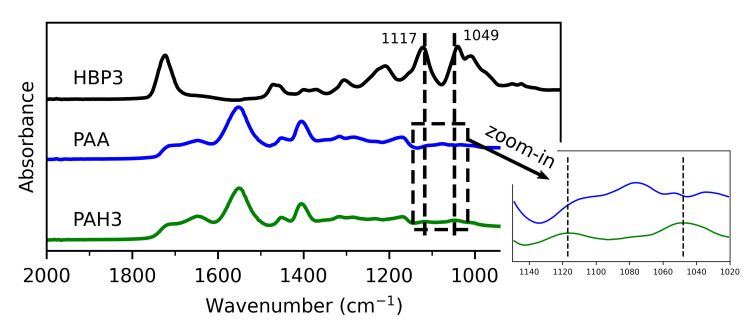
FTIR absorption spectra of the HBP3 powder (black), freeze-dried PAA hydrogel (blue), and freeze-dried PAH3 hydrogel (20 wt% HBP3) (green), between 2000 and 800 cm${}^{-1}$. All the spectra were individually normalised by using the highest peak absorption of each material. Inset: zoom-in plot showing two new emerged peaks in the spectra of the PAH3 hydrogel, relative to the PAA hydrogel.

Figure 4 shows the FTIR absorption spectra for the HBP3 powder, freeze-dried PAA, and hybrid hydrogels. Compared to the PAA hydrogel, the hybrid hydrogel indeed exhibited, yet being hardly discernible, two emerging absorption peaks at 1049 cm${}^{-1}$ and 1117 cm${}^{-1}$, corresponding to a stretching vibration of C–O in the CH–OH structure from the HBP molecule, and of O–C in the ester group of the HBP molecule, respectively [51,52]. Furthermore, compared to the spectra of HBP powder, the tiny position shift (blue shift) of the new absorption peak at 1049 cm${}^{-1}$, about 9 cm${}^{-1}$, was clearly observed (also see Figure S2, ESI). This indicated the formation of hydrogen bonding in the supramolecular network of the hybrid hydrogel [53].

**Figure 5:**
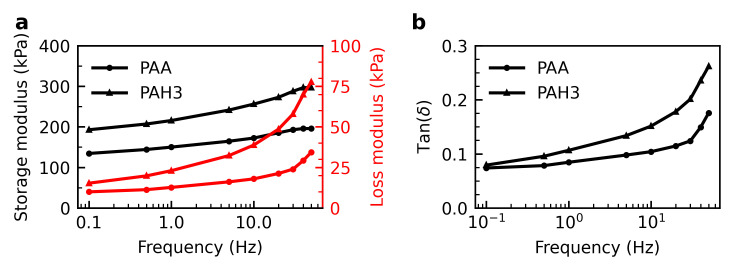
Dynamic mechanical analysis of the PAA and hybird (PAH3, 14 wt%) hydrogels under frequency sweep: (**a**) storage and loss moduli, and (**b**) loss factor tan(δ).

The responses of viscoelasticity for the PAA and hybrid hydrogels to frequency are given in Figure 5. Since weak supramolecular interactions are present by introducing HBPs into the PAA matrix, the mechanical properties of the hybrid hydrogel are anticipated to depend on the deformation rate.

For both types of hydrogels, the storage moduli were much greater than the loss moduli, confirming the solid-like behaviour of hydrogels. In DMA, loss modulus is a measure of linear viscosity and reflects relaxation time. The examined PAH3 hydrogel clearly exhibited the higher values of loss modulus than the pure PAA at high frequencies, revealing the pronounced viscous effect of introduced physical interactions through HBPs. As well, the hybrid hydrogel displayed greater tan(δ) values than the PAA hydrogel at high frequencies, indicating the potential of appreciable energy dissipation and damping, which would be further evidenced by the hysteresis measurement.

### 3.2. Mechanical Properties

**Figure 6:**
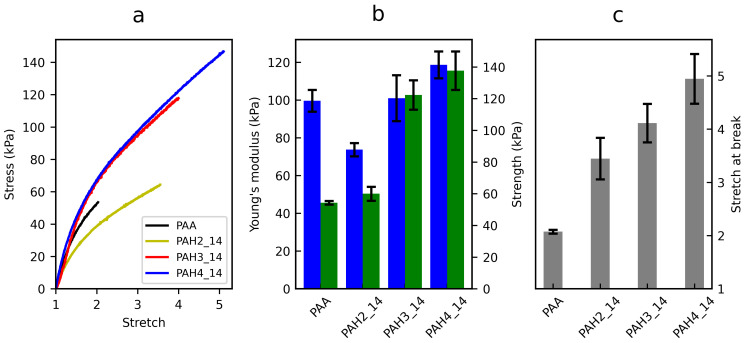
Mechanical behaviour of PAA hydrogels with and without adding HBPs, in uniaxial tension. Effects of varying the HBP generation were explored. (**a**) Typical nominal stress–stretch curves. (**b**) Young’s modulus and tensile strength. (**c**) Stretch at break. Error bars show the standard derivation (SD); sample size, n = 3 per experiment.

We explored the effects of varying the generation and content of HBP on the mechanical behaviour of the hybrid hydrogel. The strength and stretchability were greatly improved for the third and fourth generations of HBP (Figure 6b,c). When fixing the HBP content at a level of 14%, for the PAH3 and PAH4, the strengths were as high as 122.3 ± 9.3 and 137.7 ± 12.1 kPa, being 2.3 and 2.5 times that of the pure PAA hydrogel, respectively. They could be stretched to 4.1 ± 0.36 and 4.9 ± 0.47 times of the original length, increased by 100% and 140% in contrast to the PAA hydrogel. Despite the improved stretchability, the second HBP generation did not show any obvious strengthening effect. The adopted three generations of HBP possess almost the same amount of hydroxyl groups per weight (Table S1, ESI), and a similar topology (Figure 1). The second generation of HBP cannot develop as sufficient hydrogen bonding as the third and fourth generations of HBP, with the PAA network. This is likely due to small nanoparticle/aggregate size compared to the mesh size of the chemically cross-linked PAA network [54]. Another potential reason is that some end groups of hydroxyl are hidden inside the nanoparticle, as the structure of the second generation of HBP is more deformable, and more open than that of higher generations [55]. Fixing the HBP generation to the third, with increasing content of HBP3, a gradual improvement in strength and stretch at break was simultaneously found, (Figure S3b,c, ESI), revealing that more hydrogen bonds can form by tuning the content of HBPs, thus improving the mechanical behaviour.

Interestingly, the Young’s modulus remained almost unchanged at low stretch rates. Sound explanations for the seeming increase in Young’s modulus for the HBP4_14 and HBP3_20 (1.19 and 1.27 times that of PAA) are the slight effect of abundant physical cross-linking, and different water contents during tensile testing. This observation of unchanged stiffness is consistent with other experimental results [56,57]. This can be understood as follows: since the introduced hydrogen bonds by HBPs are weak in strength and fast in reforming in the PAA network, these reversible cross-links remain the load-supporting part of the molecular topology of the network mostly unchanged [58]. As a comparison, considerable enhancements of stiffness have been reported for strong and slow physical cross-links, such as sacrificial networks [14,59] and coordination bonds [60,61].

### 3.3. Fracture Toughness and Fractocohesive Length

**Figure 7:**
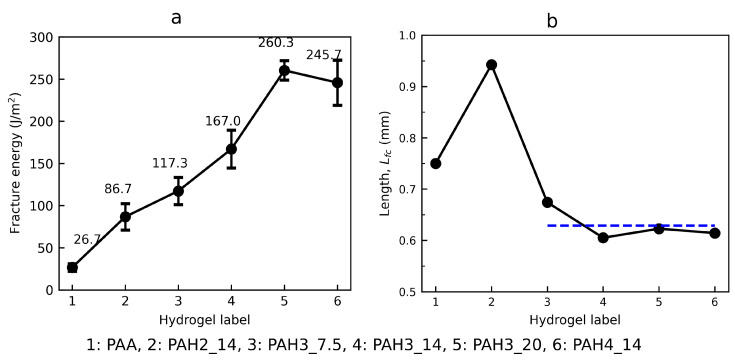
Varying fracture energy (**a**), and fractocohesive length (**b**), $L_{fc}$, as a function of hydrogel type. The hydrogel labels ranging from 1 to 6 represent PAA, PAH2_14, PAH3_7.5, PAH3_14, PAH3_20, and PAH4_14, in order. The blue dashed horizontal line in plot (**b**) shows the mean value of $L_{fc}$ among the hydrogels, labelled from 3 to 6. Error bars show the SD; sample size, n = 3 per experiment.

As expected, the toughness of the hybrid hydrogel was greatly improved relative to the pure PAA, as shown in Figure 7a. The toughness values reached 167.0 ± 22.5 and 245.7 ± 26.8 J/m${}^{2}$ for the hydrogels with the third and fourth generations of HBP at a content of 14 wt%, increased by 525% and 820%, compared to that of the pure PAA hydrogel, respectively. Furthermore, the toughness was amplified with the increase in the content of HBP3, owing to the increased density of hydrogen bonding. Despite there being little strengthening effect, the second generation of HBP still toughened the PAA hydrogel with an enhancement of 225%.

The improved toughness of the hybrid hydrogel represents a high tolerance to internal notches or cracks. To further characterise the fracture behaviour, for each hydrogel, we calculated the fractocohesive length, $L_{fc}$, measured by the ratio of fracture toughness, *G*, to work of fracture, $W_{f}$ [44]. As shown in Figure 7b, all the hydrogels displayed a fractocohesive length of a similar order of magnitude. The examined PAH_3 and PAH_4 hydrogels exhibited quite close values of $L_{fc}$, whose arithmetic mean was 0.62 mm, marked by the blue dashed line. However, $L_{fc}$ of the pure PAA hydrogel was 0.75 mm, a little larger than this mean value. We interpret this result as follows. The pure PAA hydrogel does not have any physical cross-links, and consequently the chain length and mesh size are somewhat larger than those of the hybrid hydrogel, further cross-linked by hydrogen bonds. Thereby, a slight increase in $L_{fc}$ was found for the PAA hydrogel. The fractocohesive length characterises flaw sensitivity of materials [44]. When the notch size is below $L_{fc}$, the mechanical properties are almost unaltered, whereas they are much deteriorated for the notch size beyond $L_{fc}$. A larger $L_{fc}$ typically signifies a higher resistance to notches and larger damage zone around the notch root. For our hybrid hydrogel system that incorporates HBP as a toughening agent, the $L_{fc}$ value is one order of magnitude lower than those of other remarkably tough hydrogels, such as alginate-PAAm hydrogel (∼5.2 mm) [14] and PVA-PAAm hydrogel (∼5.6 mm) [62]. Nonetheless, these tough hydrogels simultaneously exhibited considerable fracture toughness, as high as 9000 J/m${}^{2}$ and 14,000 J/m${}^{2}$, respectively, much higher than the supramolecular system in this work.

### 3.4. Hysteresis and Self-Healing

**Figure 8:**
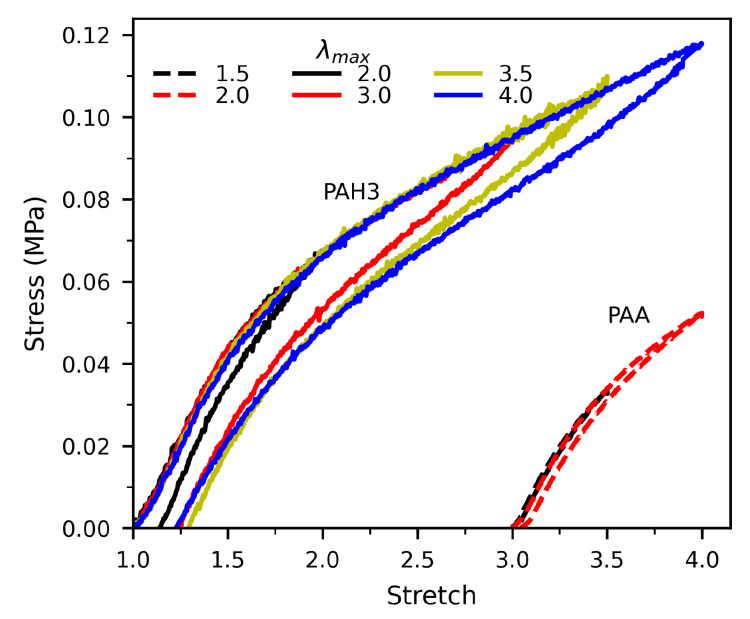
Stress–stretch curves for hydrogels of PAA and PAH3 (14% HBP3), subjected to a loading–unloading cycle with different maximum stretches, $\lambda_{max}$.

The mechanical hysteresis in soft materials is a macroscopic reflection of the breaking of the sacrificial bonds in the polymer network, during which elastic energy is dissipated [63,64]. To quantify the efficiency of energy dissipation of the hybrid hydrogel, hysteresis measurement was carried out. As shown by tensile loading–unloading curves in Figure 8, the PAA hydrogel showed little hysteresis, even at a maximum stretch close to the fracture point. However, the hybrid hydrogel with 14 wt% HBP3 underwent much pronounced hysteresis while being highly deformed. The hysteresis coefficients were 16.6%, 20.2%, and 17.5%, at the maximum stretches of 2.0, 3.5, and 4.0, respectively. In the hybrid hydrogel, the significant energy dissipation is attributed to the breaking of the formed hydrogen bonds between the PAA chains and HBP nanoparticles. With the increasing stretch, more hydrogen bonds are broken in large regions of the supramolecular network, which is evidenced by the increasing hysteresis area as in Figure 8. Since the complete reforming of broken hydrogen bonding requires a long time, at least several minutes, large residual stretches were seen for the hybrid hydrogel after unloading to zero force.

**Figure 9:**
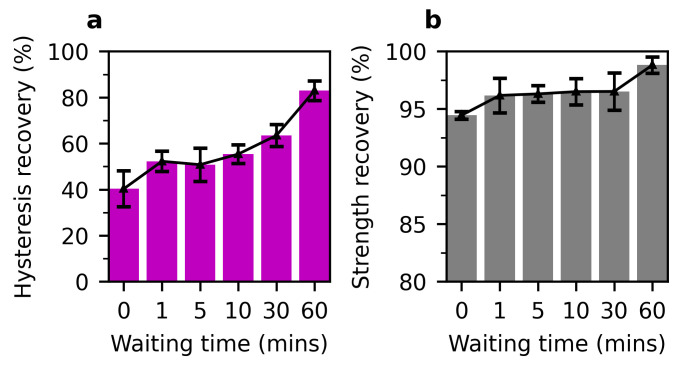
Self-healing test of the hybrid hydrogel of PAH3_14, at a maximum stretch of 3.5, and at ambient temperature. (**a**) Hysteresis recovery, and (**b**) strength recovery, as a function of waiting time. Error bars show the SD; sample size, n = 2 per experiment.

To reveal the dynamic characteristic of formed hydrogen bonding, we also examined the self-healing behaviour of the hybrid hydrogel, at ambient temperature (Figure 9). With no waiting time, the hybrid hydrogel still showed a great hysteresis recovery of 40.3 ± 7.8%, and the strength was little weakened. This is because a large fraction of broken hydrogen bonds reformed themself during slow-rate unloading. Compared to the case of no waiting, the hysteresis recovery, representing the regained capacity of energy dissipation, increased by 29.6%, even after a short resting of 1 min. After a waiting of 60 min for the stretched hydrogel, hysteresis and strength recovered to 83.0 ± 4.3%, and 98.8 ± 0.7% of the original ones. The impressive self-healing feature stems from the dynamic, reversible, yet fast nature of hydrogen bonding in this hybrid hydrogel system.

### 3.5. Mechanistic Interpretations

Hydrogen bonds play a leading role in toughening the PAA host network with HBP. The abundant carboxyl groups on the PAA chain are capable of producing numerous hydrogen bonds with the hydroxyl groups on the randomly dispersed HBP nanoparticles, as shown in Figure 2b. Despite the energy of one hydrogen bond being low [65], largely increased inter-molecular hydrogen bonds per volume can amplify toughness to a considerable extent. In addition, the dendritic architecture of HBP supermolecules increases the chance of frictional contact among polymer chains by attracting them closer by weak interactions, thus enhancing overall strength and toughness. Herein, it is derived from minor modifications in stiffness that the degree of physical entanglement is unlikely to be intensified between long PAA chains, despite the supramolecular structure of HBP [66]. Thus, hydrogen bonding should be the primary contributing factor for the toughness enhancement.

The low toughness of the pure PAA hydrogel can be well predicted by the classical Lake–Thomas model [67,68]. Due to lacking effective energy dissipation mechanisms around the crack tip, the crack can readily propagate by scissoring polymer chains laying across the crack plane. In the case of the hybrid hydrogel, however, supramolecular interactions, namely, hydrogen bonds, are able to shield the crack tip from the remote applied force, thus delaying crack propagation and improving toughness. The crush of these particular physical interactions leads to viscoelastic energy dissipation, manifested by mechanical hysteresis, through the viscoelastic deformation process in the bulk around the crack tip. The extra amount of viscoelastic energy dissipation can be roughly estimated as the fracture toughness of a hybrid hydrogel minus that of the pure PAA hydrogel.

As described previously, the third and fourth generations of HBP are more effective in toughening than the second one, since they feature adequate capacity of viscoelastic energy dissipation by introducing sufficient hydrogen bonding. Furthermore, the difference in toughening effects for the third and fourth generations can be ascribed to the hydroxyl groups that HBP nanoparticles can expose on the outer shell. Generally, a HBP of a higher generation, featuring a globular structure, should have a surface with a higher density of reactive end groups [69], and consequently gives rise to strong interactions and impacts [35,70]. The profile of HBP nanoparticles in the hydrogel might differ depending on the size and deformability of HBP molecules and in turn generation.

**Figure 10:**
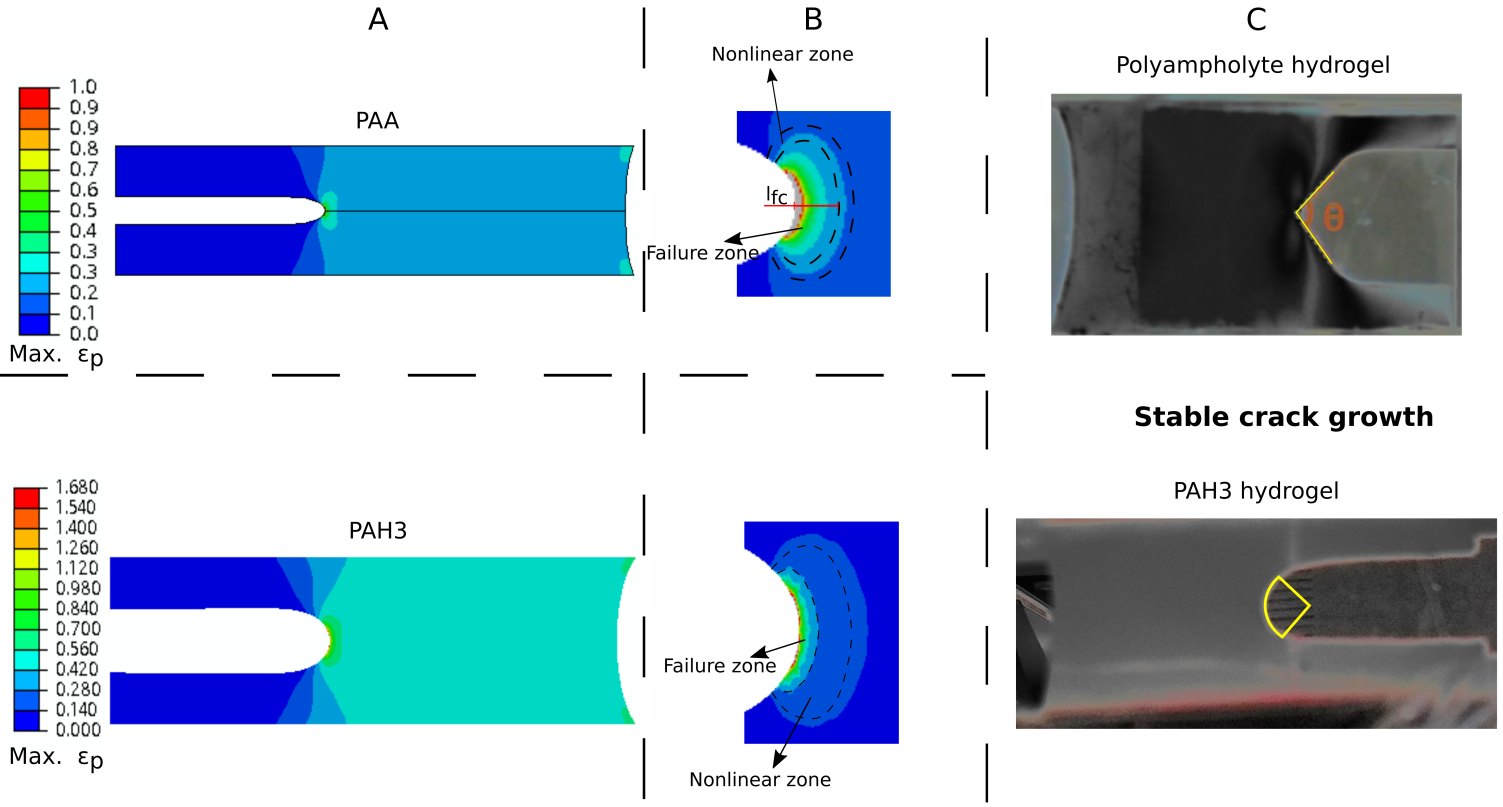
Fracture details for hydrogels containing an initial long notch, under pure shear test. (**A**) Distributions of maximum principal strain (true strain), $\epsilon_{p}$, for PAA (top) and PAH3 (14 wt%) (bottom), at the moment of crack initiation. (**B**) A zoom-in on the crack tip region. The contours correspond to the elastic strain energy density distribution. Three regions are separated by black dashed lines. (**C**) Photographs of steady-state crack growth in the notched samples, made of (top) a polyampholyte hydrogel, adapted from the work by Luo et al. [43] with permission, and (bottom) PAH3 hydrogel in this work. Note that the original photographs in C have been converted to greyscale for better visualisation.

Next, we discuss the crack-tip blunting scenario and fracture process in our hydrogels, subjected to large deformation. Owing to the pure shear configuration and material incompressibility, the deformation is subjected to the pure-shear tension in the region far from the crack tip, whereas pure uniaxial tension is the main deformation mode around the crack tip [71]. As revealed in Figure 10A, in contrast to the pure PAA hydrogel, due to amplified energy dissipation, the hybrid hydrogel displayed more pronounced blunting that is associated with large deformation, at the crack tip. Until now, there is no definitive expression for determining the size of failure zone, where damage, cracking, and healing events take place. Therefore, here we assumed that this size is equivalent to the fractocohesive length, without considering a scaling factor [71]. In both failure and nonlinear elastic zones, the polymer chains are highly stretched compared to the region far from the crack tip. In particular, in the inner failure zone, work of fracture is approaching at the onset of crack initiation (Figure 10B), and significant energy dissipation occurs. For the pure PAA hydrogel, upon initiation, the short chains break just before the long chains, contributing to energy dissipation of a small amount. For the hybrid hydrogel, however, additional physical bonds are broken in the failure zone, leading to pronounced energy dissipation. It should be noted that the size of the failure zone is typically quite small, relative to the characteristic length of the hydrogel sheet [72], and also, in general, less than the size of real energy dissipation zone in the bulk for a tough hydrogel. After crack initiation, a stable crack propagation was observed during testing, accompanied by a horizontal translation of crack-tip shape forward, which is near circular (Figure 10C, bottom). Interestingly, a triangular shape was observed for a tough polyampholyte hydrogel by Luo et al. [43] (Figure 10C, top). This difference could be explained by the high stiffness and toughness in their hydrogel. Besides the translational invariance in the crack-tip shape, in the pure shear test, the deformation and stress also hold such an invariance, whose states remain unchanged in a coordinate system moving with the propagating crack tip [48]. As emphasised in [71], two distinct physical length scales associated with the failure and nonlinear elastic zones play key roles in the fracture process of highly deformable soft materials.

Currently, reliable and accurate material constitutive models for tough hydrogels under large deformation still remain scarce, which strongly impedes the direct application of mature numerical methods to quantify stress and energy dissipation. However, these details around the crack tip are quite important for the quantitative understanding of polymer damage and fracture processes, motivating the development of innovative experimental techniques, including scission-based mechanophores [64,73], and mechanoradical polymerisation [72].

## 4. Conclusions

In summary, we have studied in detail mechanics of a supramolecular nanoparticle hydrogel system reinforced with hydroxyl-terminated hyper-branched polymers. We compared the effects of three generations of HBPs, 2, 3 and 4, on mechanical properties of the hybrid hydrogel. We found that the third and fourth generations of HBP are more effective than the second one, in terms of enhancing the strength and toughness of the PAA matrix. Specifically, at a HBP content of 14 wt%, compared to that of the pure PAA hydrogel, strengths of the hybrid hydrogels with the third and fourth HBPs are 2.3 and 2.5 times; toughnesses are increased by 525% and 820%. However, for the second generation, strength is little improved and toughness is increased by 225%. For such a discrepancy, we provided potential explanations associated with the amount of hydroxyl groups that HBP nanoparticles can expose on the outer shell.

As well, we revealed that (i) the introduced hydrogen bonds should serve as weak cross-links, since they did not contribute to the stiffness; (ii) due to the reversible, and fast nature of the hydrogen bonding, excellent self-healing was observed for the hybrid hydrogel. The toughness enhancement is ascribed to viscoelastic dissipation primarily associated with the kinematics of hydrogen bonds.

We also interpreted the fracture process by experimental observations and finite element modelling. Two critical zones around the crack tip were emphasised, where the inner failure zone is in some sense connected with the fractocohesive length. In this hybird hydrogel system, the fractocohesive length, being 0.62 mm, is one order of magnitude less than those of some remarkably tough DN hydrogels. This relationship between the fractocohesive length, and the size of the two critical zones, and other relevant characteristic lengths at different length scales is worth further investigation in order to promote the understanding of mechanics of tough hydrogels built upon supramolecular chemistry. The combination of self-healing, high stretchability, strength and toughness, yet low stiffness, makes this supramolecular hydrogel system a promising material of choice for strain sensors, capable of monitoring the deformation of soft actuators and human skins. 

## Data Availability

The data presented in this study are available on request from the corresponding author.

## Supplementary Materials

The following are available at https://www.mdpi.com/2073-4360/13/5/805/s1, Figure S1: Stress-stretch curves of the PAA hydrogel (a), and hybrid hydrogel of PAH3_14 (b), measured from experiments and fitted with the Neo-Hookean, and Mooney-Rivlin models in Abaqus, respectively. For the PAA and hybrid hydrogels, the initial shear modulus obtained from fitting was 31.96 kPa, and 40.63 kPa, respectively, Figure S2: Normalised FTIR spectra for HBP powder, PAH2_14, PAH3_14, and PAH3_20, in the range of 1000–1200 cm${}^{-1}$. The blue shift confirmed the formation of hydrogen bonding in the hybrid hydrogels with the second and third generations of HBP (Hobza and Havlas, 2000). However, by careful inspection, the peak for the second generation was a little narrower than the other two cases, likely suggesting hydrogen bonding was not as much as that corresponding to the third generation, Figure S3: Mechanical behaviour of PAA hydrogels with and without adding HBPs, in uniaxial tension. Efects of varying the HBP3 content were explored. a, Typical nominal stress-stretch curves. b, Young’s modulus and tensile strength. c, Stretch at break. Error bars show the standard derivation (SD); sample size, n = 3 per experiment. Table S1: Details for HBP molecules of different generations.
