# Supplementary material for: Mechanical Properties of a Supramolecular Nanocomposite Hydrogel Containing Hydroxyl Groups Enriched Hyper-Branched Polymers
Source: Polymers (Basel). 2021 Mar 6;13(5):805. doi: 10.3390/polym13050805 (PMC7961438; doi:10.3390/polym13050805)
Supplement: Supplementary file 1 [file polymers-13-00805-s001.pdf]

Electronic Supplementary Information:

Mechanical properties of a supramolecular hydrogel containing hydroxyl groups enriched hyper-branched polymers

Wenjin Xing,[a,b] Amin Jamshidi Ghahfarokhi,[a,b] Chaoming Xie,[c] Sanaz Naghibi,[a,b] Jonathan A. Campbell,[b] and Youhong Tang *[a,b]

According to Table S1, for the same mass of hyper-branched polymers, the hydroxyl content ratio between the three generations is 1 : 0.97 : 0.96, indicating almost the same -OH content per mass.

Table S1: Details for HBP molecules of different generations.

Generation	Formula	Molecular weight (g/mol)	No. of -OH per mass ($\times 10^{-3}$mol)
2	$C_{75}H_{128}O_{45}$	1749.8	9.1
3	$C_{155}H_{256}O_{93}$	3607.6	8.9
4	$C_{315}H_{512}O_{189}$	7323.3	8.7

For an incompressible soft material, in three dimensions, the Neo-Hookean model can be expressed as (Holzapfel, 2000)

$$\widetilde{W} = \frac{\mu}{2}(\lambda_1^2 + \lambda_2^2 + \lambda_3^2 - 3) \tag{1}$$

where $\widetilde{W}$ denotes the strain energy density, μ is the shear modulus, and λ_i the $i-$th principle stretch (i=1,2,3).

[a] *College of Science and Engineering, Flinders University, Clovelly Park, Adelaide, SA 5042, Australia*

[b] *Institute for NanoScale Science and Technology, Flinders University, Bedford Park, Adelaide, SA 5042, Australia*

[c] *Key Lab of Advanced Technologies of Materials, Ministry of Education , School of Materials Science and Engineering, Southwest Jiaotong University, Chengdu, Sichuan, 610031, China*

In three dimensions, the Mooney-Rivlin incompressible model can be expressed as (Holzapfel, 2000)

$$\widetilde{W} = C_1(\lambda_1^2 + \lambda_2^2 + \lambda_3^2 - 3) + C_2(\lambda_1^{-2} + \lambda_2^{-2} + \lambda_3^{-2} - 3) \tag{2}$$

where the shear modulus μ can be computed as $\mu = 2(C_1 + C_2)$. Note that the Neo-Hookean material can be regarded as a special case of the Mooney-Rivlin model, when $C_2 = 0$.

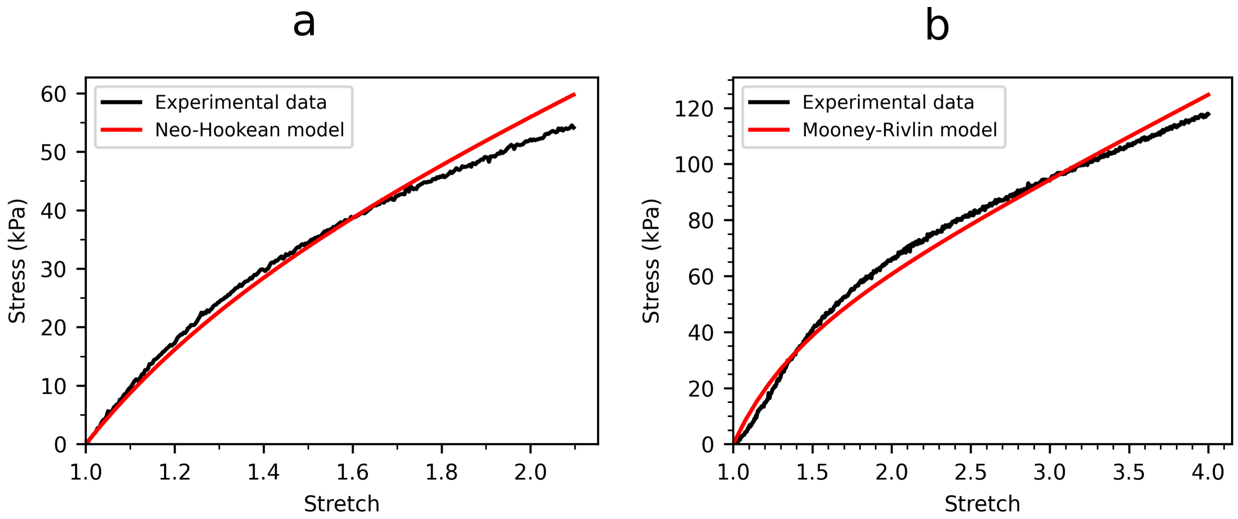

Figure S1: Stress-stretch curves of the PAA hydrogel (a), and hybrid hydrogel of PAH3_14 (b), measured from experiments and fitted with the Neo-Hookean, and Mooney-Rivlin models in Abaqus, respectively. For the PAA and hybrid hydrogels, the initial shear modulus obtained from fitting was 31.96 kPa, and 40.63 kPa, respectively.

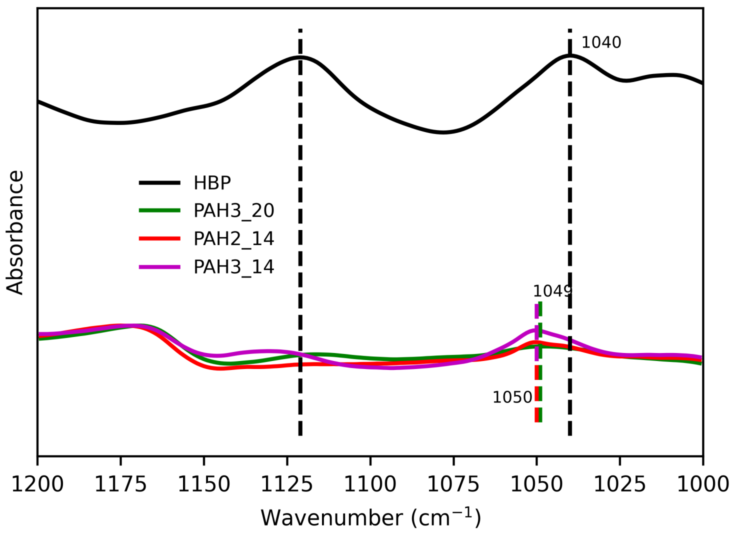

Figure S2: Normalised FTIR spectra for HBP powder, PAH2_14, PAH3_14, and PAH3_20, in the range of 1000-1200 cm^{-1}. The blue shift confirmed the formation of hydrogen bonding in the hybrid hydrogels with the second and third generations of HBP (Hobza and Havlas, 2000). However, by careful inspection, the peak for the second generation was a little narrower than the other two cases, likely suggesting hydrogen bonding was not as much as that corresponding to the third generation.

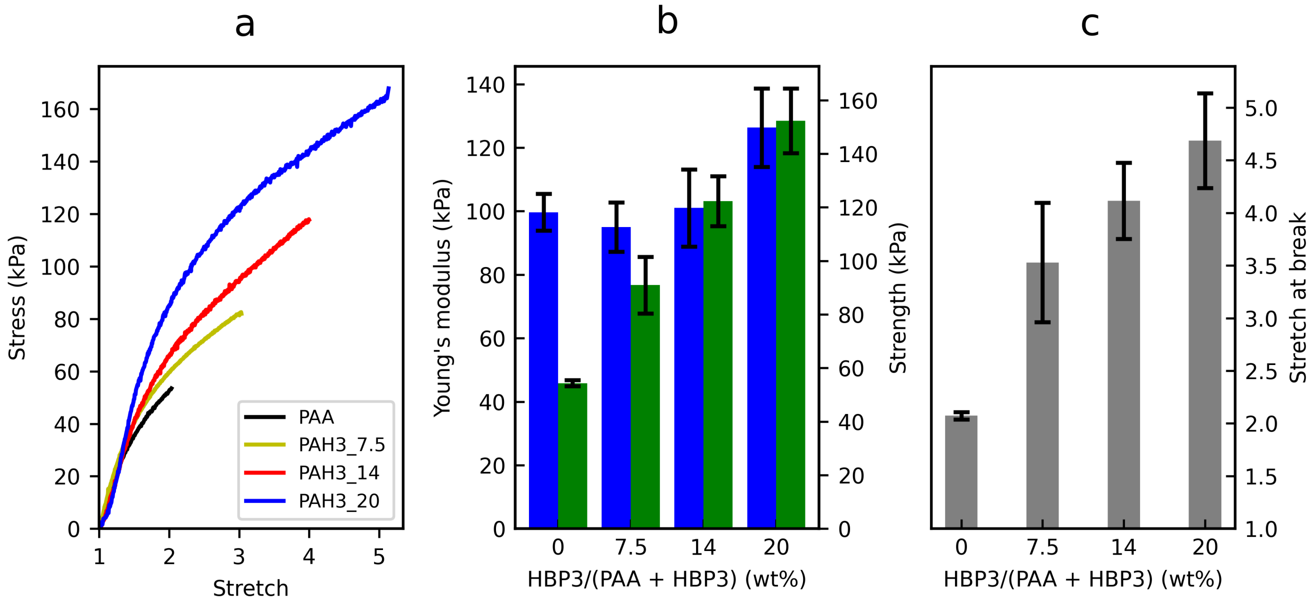

Figure S3: Mechanical behaviour of PAA hydrogels with and without adding HBPs, in uniaxial tension. Effects of varying the HBP3 content were explored. a, Typical nominal stress-stretch curves. b, Young's modulus and tensile strength. c, Stretch at break. Error bars show the standard derivation (SD); sample size, n = 3 per experiment.

References

[1] Hobza, P. and Havlas, Z. (2000). Blue-shifting hydrogen bonds. *Chemical reviews*, 100(11):4253–4264.

[2] Holzapfel, A. G. (2000). Nonlinear solid mechanics: a continuum approach for engineering.
